# Real-time respiratory motion prediction using photonic reservoir computing

**DOI:** 10.1038/s41598-023-31296-2

**Published:** 2023-04-07

**Authors:** Zhizhuo Liang, Meng Zhang, Chengyu Shi, Z. Rena Huang

**Affiliations:** 1 Rensselaer Polytechnic Institute, Troy, NY 12180 USA; 2 City of Hope Medical Center, Duarte, CA 91010 USA

**Keywords:** Radiotherapy, Computer science, Optoelectronic devices and components

## Abstract

Respiration induced motion is a well-recognized challenge in many clinical practices including upper body imaging, lung tumor motion tracking and radiation therapy. In this work, we present a recurrent neural network algorithm that was implemented in a photonic delay-line reservoir computer (RC) for real-time respiratory motion prediction. The respiratory motion signals are quasi-periodic waveforms subject to a variety of non-linear distortions. In this work, we demonstrated for the first time that RC can be effective in predicting short to medium range of respiratory motions within practical timescales. A double-sliding window technology is explored to enable the real-time establishment of an individually trained model for each patient and the real-time processing of live-streamed respiratory motion data. A breathing dataset from a total of 76 patients with breathing speeds ranging from 3 to 20 breaths per minute (BPM) is studied. Motion prediction of look-ahead times of 66.6, 166.6, and 333 ms are investigated. With a 333 ms look-ahead time, the real-time RC model achieves an average normalized mean square error (NMSE) of 0.025, an average mean absolute error (MAE) of 0.34 mm, an average root mean square error (RMSE) of 0.45 mm, an average therapeutic beam efficiency (TBE) of 94.14% for an absolute error (AE) < 1 mm, and 99.89% for AE < 3 mm. This study demonstrates that real-time RC is an efficient computing framework for high precision respiratory motion prediction.

## Introduction

A reservoir computer (RC), often referred to as an echo-state network^1^, is one class of recurrent neural networks (RNN) that is rather efficient at processing temporal or sequential signals. The neurons (nodes) in the hidden layer of an RC have random but fixed connections, while only the readout layer weights are trained using computationally inexpensive linear regression methods^2,3^. Compared to machine learning frameworks of iterative learning, an RC is an appealing artificial neural network (ANN) approach for its simplicity in network architecture and algorithm implementation, and thus fast computation capability.

To date, RC has been applied to both benchmark and real-life computation tasks such as pattern classification^4,5^ and generation^6^, time series forecasting^7^, voice recognition^8^, equalization for a wireless channel^9–11^, satellite communication^12^, and so on. Recently, a photonic RC has been used for optical signal recovery in fiber communication that requires ultra-fast signal processing speed to classify highly distorted optical signals^13^. In this task, the optical signal waveform is distorted by multiple sources of nonlinear perturbations along the fiber. A common theme here is that the perturbations, though intense and nonlinear, are applied constantly to the traveling signals so the waveform distortion is repeatable when the signals go through the same fiber channel. There exists another class of problems, little studied so far, in which the physical perturbations to the signals are stochastic, resulting in non-repeatable waveforms. Living biological systems often behave in this manner and can display greatly varying amplitudes at random. Such extreme signal distortion and variety complicate the ability of RC to recognize and classify signal patterns.

In the medical field, respiration induced inner organ/tumor motion provides an example of an arbitrary temporal signals where the tumor follows complex, quasi-sinusoidal waveform. The respiratory motion is intrinsically dynamic with continuously changing characteristics in any interval of time, punctuated by occasional deviations caused by involuntary events, such as sneezing and coughing, that cause large variations in displacement amplitude and frequency. Respiratory motion is a well-recognized issue for chest and upper abdominal body imaging and radiotherapy for cancer patients suffering from lung, liver, throat, stomach, or pancreatic cancers. Extensive studies and several clinical approaches have been reported in managing the respiratory tumor motions^14,15^.

For adaptive radiation therapy, the radiation beam is repositioned in real-time according to the respiratory motion so that the beam always remains on-target. Several machine learning methods including feed-forward neural networks (FNN), recurrent neural networks (RNN), and convolutional neural networks (CNN) have been explored for respiratory motion prediction^16–19^. These existing approaches generally require a large cohort of patient datasets for network training. For example, one study utilized 143 time series^20^ while another used 306 time series each having 100,000 data points for network training^21^. Neural network training time is another challenge in learning-based respiratory motion prediction. One group reported training times > 12 h using a temporal CNN^22^. RNNs are generally thought to be better suited for temporal signal processing with feedback from earlier inputs; however, classic RNNs are notoriously difficult to train^23^. Long-short term memory (LSTM) networks have been used in respiratory motion prediction by several groups^21,24^. Lin et al.^24^ reported training times > 20 h over hundreds of breathing curve time series, while Mafi et al.^21^ reported training times around 10 h on 100 time series of data.

The respiratory motion signals are quasi-periodic, nonstationary data and forecasting the tumor motion trajectory in real-time is essential for clinical practice. In this work, we present a photonic RC approach that provides a solution for real-time respiratory motion prediction. Predictions made through this approach can be completed within the permitted look-ahead time, i.e. the latency necessary for adapting the radiation modality for beam size/shape/location adjustment during a procedure, demonstrating its suitability for real-time adaptive radiotherapy treatment. The proposed RC algorithms can be trained with very minimal data, thus substantially reducing the neural network training complexity while producing comparable prediction accuracies to other more computational demanding machine learning algorithms^18–24^.

The reservoir layer of the RC is implemented in a true-time delay photonic circuit constructed from off-the-shelf commercial components. And it can be further realized on chip-scale Si platform^25–27^. In this work, we studied a double-sliding window data processing strategy in which the input dataset window and the training dataset window slide in sync to process the livestreamed tumor position data without discernable latency. The major contributions of this study are summarized as follow. (1) The training dataset has only 600 data points (20 s of data) for fast algorithm execution, while sliding the training dataset ensures that the weight matrix is trained only on the latest respiration signals while discarding distant ones. The RC processing time, including RC network training and prediction calculation, is projected on the order of tens of milliseconds. An enhanced precision in the predicted position of the tumor is thus obtained in real-time as compared with predictions obtained with an RC network trained on a fixed dataset. Unsynchronized photonic RC is implemented to increase the dynamics of the reservoir and reduce the reservoir state vector dimension in favor of fast computing. (2) It is shown that tumor motion can be precisely predicted using an algorithm based on a photonic true-time delay reservoir (TDR) computer. (3) It is shown that such a RC computing algorithm can yield a generalized processing approach for a class of nonstationary temporal signals characterized by stochastic perturbations and a rich diversity of waveform distortions.

The article is organized as follows: the theoretical background of delay-line RC network and the experimental setup used in this work are introduced in the “Methods” section. It then is followed by the detailed discussion on the real-time RC algorithms explored in this work. The RC prediction results of all 76 patients are presented in the “Results” section. The result analysis and discussion are presented in the “Discussion” section and then followed by a “Conclusion and future works” section.

## Methods

The tumor respiratory motion data is collected from an external surrogate using a Varian system (RPM, Varian Medical Systems, Palo Alto, CA, USA). In this study, the respiratory motion dataset consists of 76 pieces of breathing patterns in varying durations from 1 to 4 min. The sensor sampling rate is 30 Hz. The radiator system latency in adjusting the multi-leaf collimator is caused by the combined software and hardware delay. In this work for the purpose of general study, we focus on short to medium range of equipment latency, i.e., the motion prediction look-ahead time of 66.6, 166.6, and 333.3 ms.

All respiratory motion data in this research were acquired in accordance with clinical written policies and procedures about respiratory motion control. Patients had signed an informed consent for the clinical treatment. The Cancer Therapy and Research Center, San Antonio, TX, Institutional Review Board (IRB) approved the research under an NIH grant agreement. All the data was further anonymized to share with the research collaborator. The approach of incorporating the photonics RC for motion prediction in adaptive radiation therapy is discussed in the supplement.

### Delay-line RC network

**Figure 1:**
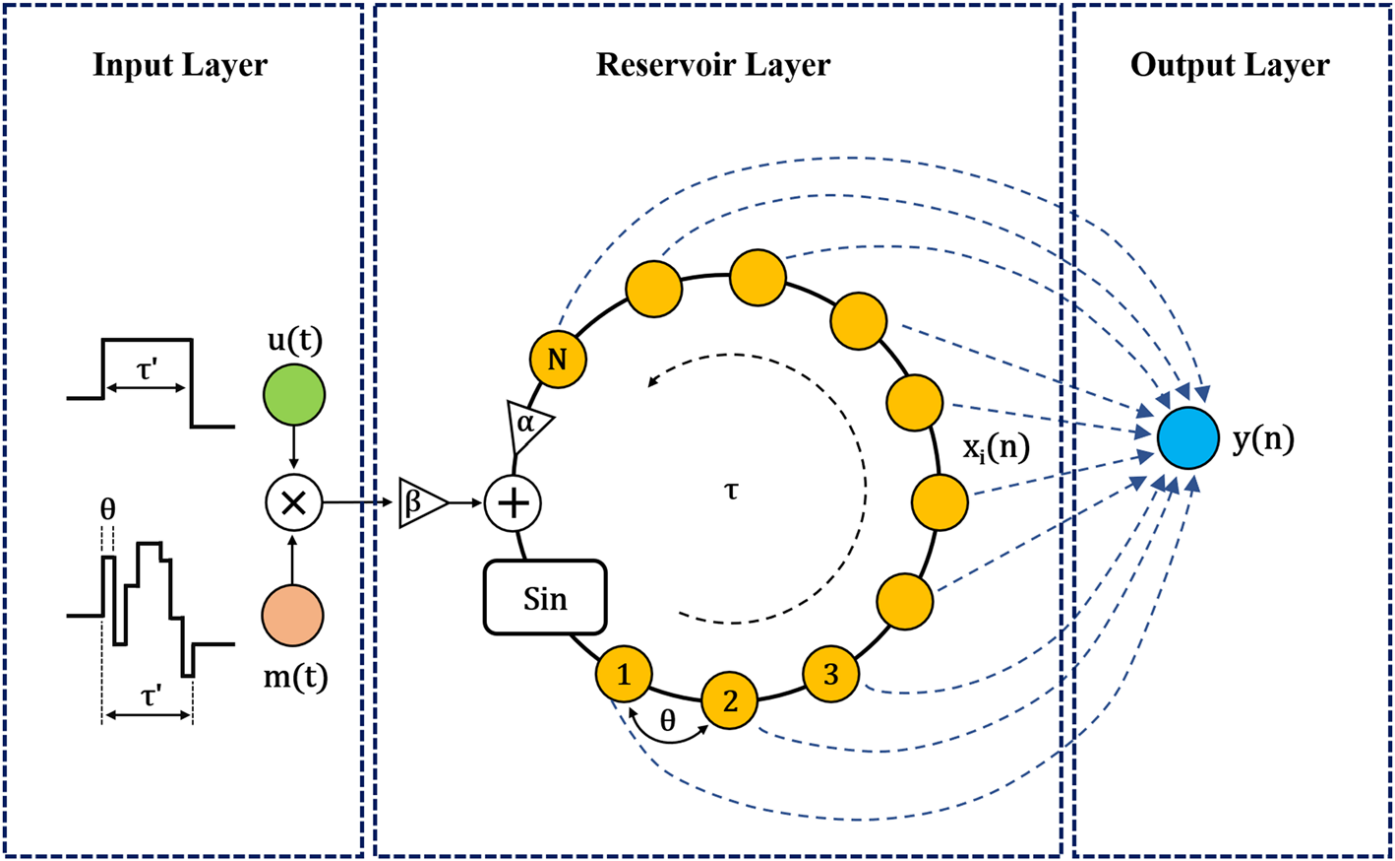
A schematic representation of a photonic TDR topology. A total of *N* virtual nodes are connected in a closed-loop ring architecture. The input signals are projected to the reservoir via a mask function while a gain *β* is set for the coupling strength. The nonlinear node, Sin, provides a sinusoidal function with an internal loop signal amplification factor (gain) $\alpha$.

Several RC topologies have been reported based on how the neurons in the hidden layer are connected^28,29^. Here, we implement a hidden layer delay line topology with adjustable connections between nodes in a fiber-optic true-time delay implementation. A schematic representation of the RC network is shown in Fig. 1. This RC network consists of three layers: input, reservoir, and output layer. The reservoir layer is implemented online with the true-time delay photonic circuit discussed here. At this stage of research, the input and output layer of the RC are processed offline, but RC can be implemented in real-time by using photonic hardware signal processing^30,31^. A single nonlinear physical node is utilized to map and project the input layer signals to the reservoir layer. A large number of virtual nodes are stored temporally along a delay line. A position sensor records and sends digitized sequential motion signals every ${t}_{s}$ = 33.3 ms. The digitized input signal $u(n)$ undergoes a sample-and-hold operation of duration *τ*′ to produce a continuous function $u(t)$, where *n* is the motion data index and *τ*′ is user defined. The input signal $u(t)$ is then projected to the reservoir layer by multiplying with a mask function $m\left(t\right)$ with a periodicity identical to *τ*′. The piecewise mask function levels are drawn from a random distribution such that the duration of each piece, *θ*, and the reservoir’s number of virtual nodes, *N*′, satisfies *θ* = *τ*′/*N*′. The photonic RC processes continuous analog signals that have the form of

1 $$u\left(t\right)=u\left(n\right), n{\tau }^{\prime}\le t<(n+1){\tau }^{\prime}$$

2 $$m\left(t\right)={m}_{i}, n{\tau }^{\prime}+i\theta \le t<n{\tau }^{\prime}+(i+1)\theta$$

where $i$ represents the *i*th node in the delay line. The mapping of low dimensional input data to a reservoir state in a high dimensional dynamic space greatly enhances the separability of classes of data^32^. The reservoir state values $x\left(t\right)$ are impacted by both the current inputs and the past inputs, and can be described by an evolution equation:

3 $$x\left(t\right)= sin\left(\alpha {W}_{res}x\left(t-{\tau }^{\prime}\right)+\beta m(t)u\left(t\right)+\varphi \right),$$

where ${W}_{res}$ is the reservoir internal connectivity; and *α*, *β* and *φ* are the hyperparameters of the RC that need to be optimized per computation task. The delay line in the reservoir forms a closed loop, similar to a feedback loop or an optical oscillator cavity^33^. The round-trip delay, i.e., the oscillator characteristic time, denoted by $\tau$, is determined by the combined optical and electrical signal travelling time in the loop. The reservoir state can be discretized by sampling a single point on the delay line, with a period $\theta$, to produce *N*′ pieces of nonlinear response, ${x}_{i}\left(n\right)$, to each input data $u(n)$. The dynamics of the reservoir for a typical synchronized RC operation^5^, i.e., *τ*′ = *τ*, can be described by discretized reservoir states $x\left(n\right)$ where $x\left(n\right)= {[{x}_{1}\left(n\right)\dots {x}_{i}\left(n\right)\dots {x}_{{N}^{\prime}}\left(n\right)]}^{T}$ and each element is obtained as follow:

4 $${x}_{i}\left(n\right)= sin\left(\alpha {x}_{i}\left(n-1\right)+\beta {m}_{i}u\left(n\right)+\varphi \right),$$

An optical Mach–Zehnder modulator (MZM) is used as the nonlinear node and provides the sinusoidal transfer function. Mathematically, other nonlinear functions, such as hyperbolic tangent function can also be used^34^ for the RC. The projected signals in the reservoir travel unidirectionally in the delay-line loop and the optical power is attenuated until the signal strength is reduced below the noise level, emulating short-term memory of a biological neural system, which will be referred to in this work as the decaying memory. The target output, i.e., the ground truth, is marked as $y(n)$ and ${{{\widehat{y}}}}\left(n\right)$ is the predictive value. The output weight matrix ${W}_{out}$ is calculated via ridge regression^30^ during the training process:

5 $${W}_{out}=Y{X}^{T}{(X{X}^{T}+\lambda I)}^{-1},$$

where the subscript *T* denotes the matrix transpose operation, $\lambda$ is the regularization factor typically very small ($\lambda =0.01$ used in this work), $Y$ is the target output matrix, $X$ is the reservoir state matrix (${\mathbb{R}}^{{\mathrm{N}}^{\prime} \times {k}_{tr}}$) and $I$ is the identity matrix (${\mathbb{R}}$^N′×N′^). The most computationally expensive step is matrix inversion operation. Once ${W}_{out}$ is determined in the training process, the predicted ${{\widehat{y}}}\left(n\right)$ is the linear sum of the weighted reservoir output neurons:

6 $${\widehat{y}}\left(n\right)={W}_{out}\cdot x\left(n\right).$$

### Experimental setup

**Figure 2:**
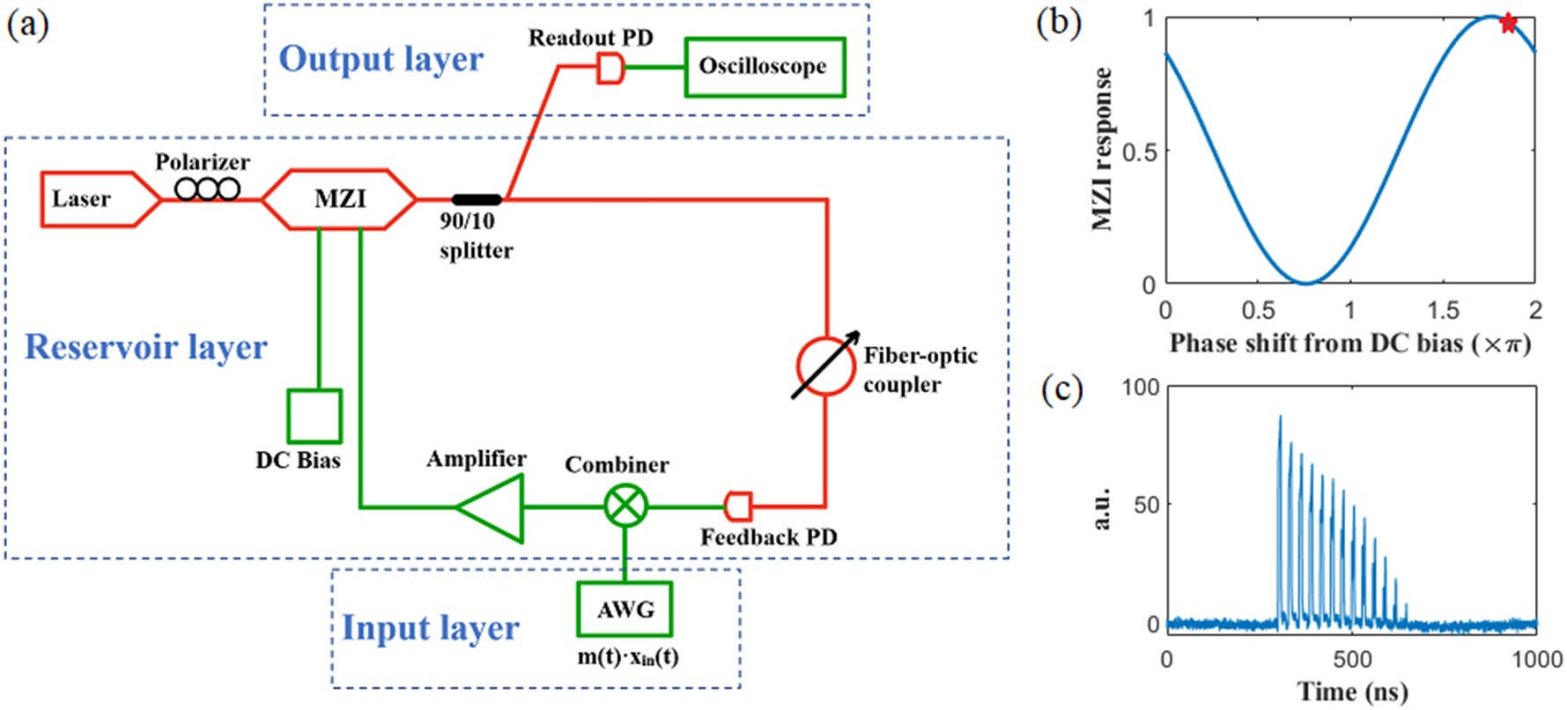
(**a**) Schematic of photonic real-time delay-line reservoir system. (**b**) Transfer function and operating point (marked as a red star) of the MZM (**c**) Spike train characterizing a decaying memory pattern^5^.

The real-time RC algorithm is implemented on a bench-top photonic TDR system constructed from off-the-shelf fiber optical components. A schematic representation of the TDR is shown in Fig. 2. The nonlinear node of the RC is realized by an optical amplitude modulator (EOSPACE AX-0MSS-20-PFA-SFA-LV). Adjusting the DC bias, the operation point of the modulator is set at 0.05 $\pi$ right to the peak of the sinusoidal transfer function, giving $\varphi$ = 0.55 $\pi$ in Eq. (3). Besides several short fibers used to connect various optical components, there is no dedicated fiber used for extra time delay. The measured intrinsic time delay of the photonic TDR is 28 ns which comprises the true-time delay needed for the reservoir computer. A fiber-optic coupler (Newport F-CPL-1550-N-FA) and an electrical amplifier (Tektronix PSPL5865) are used in the TDR, the combining of which controls the feedback gain α.

It is worth noting that the electrical amplifier makes it easier to adjust the gain *α*, but it is not a must-have component and can be excluded in the photonic RC. The input gain $\beta$ is the coefficient of *u(t)* generated by an arbitrary waveform generator (AWG) (Keysight M8190), so it is only affected by the electrical portion of the delay loop (green path in Fig. 2a). The feedback gain $\alpha$ is determined by both the optical path (red path in Fig. 2a) and the electrical path. The spike train method^35^ presents visualization of the decaying memory of the RC and provides a simple approach to characterize how sensitive the reservoir dynamics in response to external stimuli. The decaying memory illustrates the memory of RC and enables the rich dynamics in the system^36,37^. A decaying ratio is defined by the signal intensity between two consecutive pulses. In this work, a decay ratio of 0.87 is set in Fig. 2c. The decaying ratio can also be treated as a hardware based hyperparameter and optimized for one task. The extracted feedback gain $\alpha$ and $\beta$ values are $\alpha =0.87$, $\beta =2.29$ based on measurement results. Details of $\alpha$ and $\beta$ calculation are described in the Supplementary material. The pulse train shown in Fig. 2c indicates that the input signals are retained in the TDR with an attenuated amplitude, emulating the short-term memory of biological systems. The RC possesses temporal interactions among reservoir states of the different nodes as shown in Eqs. (3–4).

In this study, the cyclic delay-line loop has a round-trip characteristic time of $\tau$ = 28 ns and the piecewise function duration of $\theta$ = 2 ns. The input signal, i.e. the tumor position was set with a sample-hold duration $\tau^{\prime}$ = 24 ns by the AWG. The hold time *τ*′ gives rise to a virtual node number of $N^{\prime}$ = 12 in the delay loop even though the maximum available number of virtual nodes is $N$ = 14. The offset of *N*′ from *N* is to introduce asynchronization in node interaction that enriches the reservoir dynamics.

## RC algorithms for real-time tumor position prediction

Current machine learning methods often require acquisition of a minute to several minutes of motion signals to properly train a neural network for the required computational precision^22,32,38^. Clinic practice requires a maximum desirable training time of less than 30 s. This is because the radiation beam needs to be shuttered during neural network training and beam re-alignment needs to be verified. This adds overhead time to the total radiation treatment efficiency. In this work, we trimmed the training dataset length from several minutes to 20 s with improved predictive location precision in a real-time clinical setting. The training data length ${k}_{tr}$ is defined as the number of data points used for RC network training. A smaller ${k}_{tr}$ not only enhances the radiation treatment efficiency but also reduces the reservoir state $X$ dimension favoring faster RC algorithm execution.

**Figure 3:**
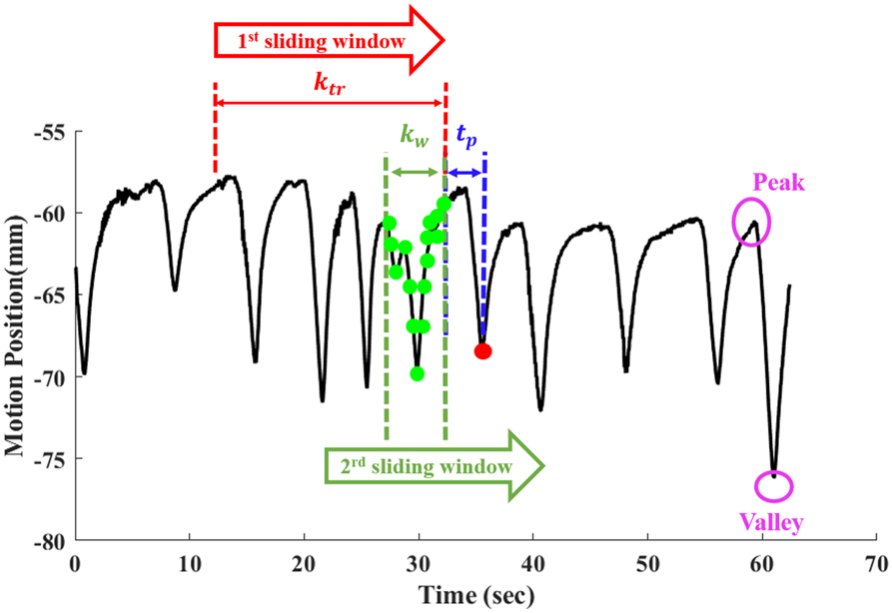
A schematic representation of the algorithm implemented in our photonic RC network for real-time tumor motion prediction. Dual-sliding windows comprising ${t}_{tr}$(20 s) and ${k}_{w}$ (15 data points) are sliding in sync. The maximum look-ahead time studied in this work is ${t}_{p}$ = 333 ms, which corresponds to ${h}_{p}$ = 10. Figure shows one prediction example, where the input window data (green dots) are used to predict the data value 333 ms in the future (red dot).

A novel dual-sliding window strategy is adopted in the execution of the algorithm, as exemplified in a small portion of a respiratory motion curve shown in Fig. 3. The two sliding windows refer to the training data window ${k}_{tr}$ and the input data window ${k}_{w}$, respectively. Both have a fixed data length, and both slide in synchronization such that the data content in both windows varies. The training data window takes on a new position while discarding the most distant data to maintain a fixed ${k}_{tr}$ length. This data refresh operation generates a new training dataset, thus establishing a new or updated ${W}_{out}$ matrix with time. This strategy enables an uninterrupted, real time, respiratory motion prediction with minimal therapeutic beam shuttering, resulting optimal beam utilization time and optimal clinical beam dose efficiency. We evaluated ${k}_{tr}$ between 300 and 1000 data points and observed that the ${k}_{tr}$ = 600, corresponding to a window length in time of ${t}_{tr}=20$ s, is sufficient to produce satisfactory motion prediction results for most breathing patterns.

The second sliding window refers to an input data window with a fixed length ${k}_{w}$, used in forecasting tumor position. A larger ${k}_{w}$ means more position data points from the past are utilized in forecasting of the next several tumor positions. Typically, a greater ${k}_{w}$ value produces better prediction results; however, for ${k}_{w}$ > 15, the increase in prediction precision is diminished so we set ${k}_{w}=15$ in processing all 76 patient-breathing curves for comparison. The input sliding window follows the same refreshing operation as the training sliding window. The look-ahead time, ${t}_{p}$ is defined as a future time point at which the respiration caused tumor movement is calculated by the RC. The look-ahead time ultimately is determined by the radiator reposition latency (see Supplementary for details). The number of data points within the look-ahead time duration is ${h}_{p}$. In this work, we explored three look-ahead times: ${t}_{p}$ = 66.6, 166.6, and 333 ms corresponding to ${h}_{p}$ = 2, 5 and 10, respectively.

We use the following set of parameters: ${h}_{p}=10$, ${k}_{tr}$ = 600, and ${k}_{w}=15$ as an example to explain how motion prediction by the real-time RC algorithm works. The first RC training model is established following by Eqs. (1–5) to produce the first ${\mathrm{W}}_{\mathrm{out}}$ matrix after processing the first 20 s of position data, i.e., $\left\{{y}_{1}\dots {y}_{600}\right\}$ in the photonic RC. When ${h}_{p}$ = 10, the first predicative data index is 610 to yield a prediction value of ${{\widehat{y}}}_{610}$ using the input data from the sliding window of ${k}_{w}$, $\left\{{y}_{586}\dots {y}_{600}\right\}$. The motion data acquired in each interval sets the time budget for signal processing, including data acquisition, signal processing in the RC input, reservoir and output layers, computation time to obtain ${W}_{out}$ and prediction calculation of ${\widehat{y}}$. All these computation steps need to finish within 33 ms as the next position data ${y}_{601}$ will be fed to the RC network to repeat the process. The training dataset ${k}_{w}$ is updated to be $\left\{{y}_{2}\dots {y}_{601}\right\}$ while a new output weight matrix ${W}_{out}$ is generated to compute ${{\widehat{y}}}_{611}$ with input data window ${k}_{w}$ changing to $\left\{{y}_{587}\dots {y}_{601}\right\}$. The process continues until the radiation therapy session stops, which means we will have tumor respiratory motion prediction data from ${{\widehat{y}}}_{610}$ until the end of a time series. For example, the final prediction data matrix could be ${\widehat{y}}=\left\{{{\widehat{y}}}_{611}\dots {{\widehat{y}}}_{3600}\right\}$ for a radiation treatment session of 2 min.

## Results

A total of 76 breath patterns were collected and analyzed for motion prediction using the photonic TDR. All patients breathing pattern data were anonymized while the motion signal curves are numbered as breath pattern (BP) 1–76. Signal noise in data acquisition and digitization is inevitable due to the electronic components used in the devices including the position sensor and other electronic instruments. A digital Gaussian filter is applied on the motion data to minimize these noise effect (see more details in Supplementary material).

**Figure 4:**
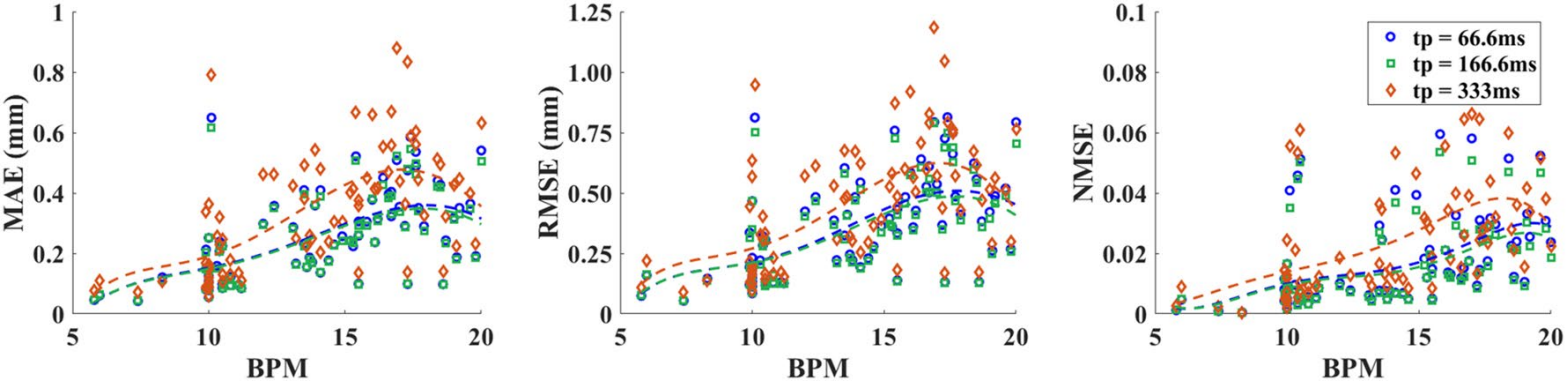
MAE, RMSE and NMSE for respiratory motion prediction of all 76 patients as a function of breathing speed (BPM), with 5th order polynomial trendlines (dashed lines). (Testing conditions: $N=14$, $N^{\prime}=12$, ${k}_{tr}=600$).

**Figure 5:**
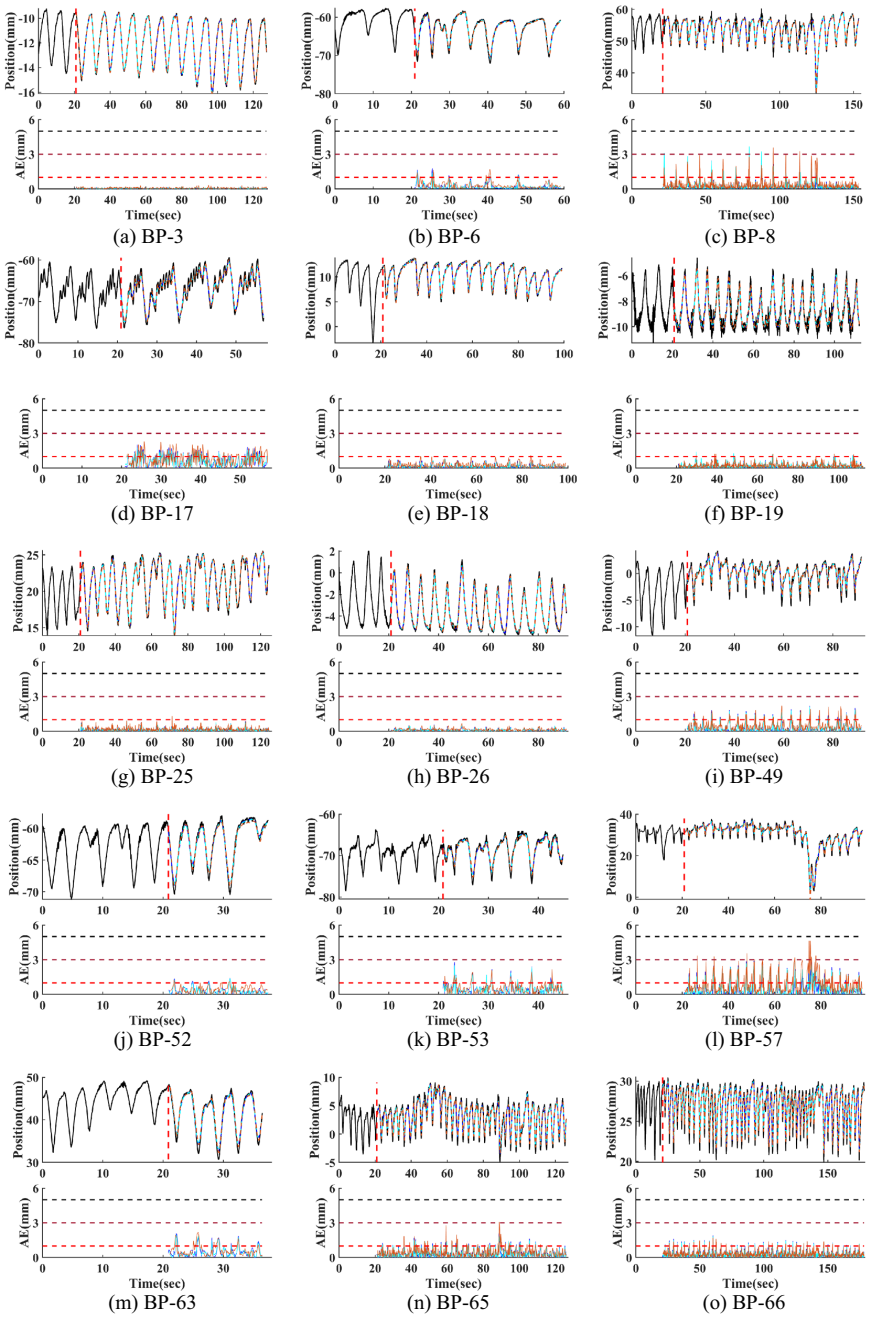
RC motion prediction results for BPM 1–10 (**a**–**d**), BPM 10–15 (**e**–**j**), BPM 15–20 (**k**–**o**). The first 20 s of the motion signals are included in the breathing pattern graphs while the first predicted motion signal occurs at $t$ = ${t}_{p}+20$ s. In the position versus time graph, the predictive values at ${t}_{p}$ = 66.6 ms (blue), 166.6 ms (cyan), and 333.3 ms (brown) are plotted with the ground truth (black) for comparison. Corresponding NMSE and MAE for each motion curve prediction are listed on top of each graph. In the AE vs time graph, the absolute error are plotted with three criteria dash lines of AE = 1 mm (red), 3 mm (brown), 5 mm (black).The irregularities of the breath patterns are summarized here: (**a**) normal (BP-3 ), (**b**) amplitude and frequency variation (BP-6), (**c**) pulse (BP-8), (**d**) shape irregularity (BP-17), (**e**) pulse (BP-18), (**f**) breathing pattern shape irregularity, i.e. inverse shape (BP-19), (**g**) baseline shift + double breathe (BP-25), (**h**) inverse-shape (BP-26), (**i**) amplitude change + baseline shift (BP-49), (**j**) double breathe (BP-52), (**k**) shape irregularity (BP-53), (**l**) pulse (BP-57), (**m**) amplitude change + baseline shift (BP-63), (n) baseline shift (BP-65), (**o**) normal (BP-66). (Testing conditions: $N=14$, $N^{\prime}=12$, ${h}_{p}$ = 10, ${t}_{p}$ = 333 ms, ${k}_{tr}=600$).

In a full breath cycle, the patient goes through inhale and exhale phases, leading to a motion curve of peak-valley-peak. Depending on the tumor location, the respiration induced tumor movement can vary over a large range^39,40^. The motion amplitude pertaining to 76 patients used in this study ranges from 5 mm to 3 cm, while the RC algorithm for motion prediction is directly applied on the raw position data without normalization. It is common that a breathing pattern of a patient combines multiple irregularities, resulting in exceedingly versatile temporal motion curves. Mathematically, temporal signals with a large degree of waveform distortion from a baseline quasi-sinusoidal function, would require much higher-class separability in the high-dimensional reservoir space to achieve the same level of prediction precision. For adults at rest, the respiration rate is normally at 12–16 BPM. At cancer radiation clinics, a wide range of BPM values, ranging from 3 to 20^41,42^ is often observed and treated by radiotherapy. The 76 breathing patterns are arranged in a sequence from low to high BPM in Fig. 4. The prediction errors are evaluated by the NMSE, MAE, and RMSE, defined as

7 $$NMSE=\frac{\sum_{i=1}^{n}{(\widehat{{y}_{i}}-{y}_{i})}^{2}}{\sum_{i=1}^{n}{(\overline{{y }_{i}}-{y}_{i})}^{2}} ,$$

8 $$MAE=\sum_{i=1}^{n}\frac{\widehat{{y}_{i}}-{y}_{i}}{n},$$

9 $$RMSE= \sqrt{\sum_{i=1}^{n}\frac{{(\widehat{{y}_{i}}-{y}_{i})}^{2}}{n},}$$

where $n$ is the total number of motion position data points predicted, $i$ represents the *i*th data point, $\widehat{{y}_{i}}$ stands for the predictive value of ${y}_{i}$ and $\overline{{y }_{i}}$ is the averaged position value of all $n$ points. All predicted motion results in one time series are included in NMSE, RMSE and MAE calculation. As illustrated in Fig. 5, the vertical red dashed line marks the first training set whereas the predicted data comprises the remaining data points after the dash line starting at data index $n={k}_{tr}+{h}_{p}$ ($n=610$ in this case). As the position sensor collects data at a fixed rate, a higher BPM implies a lower density of data points per respiration cycle. The NMSE, RMSE and MAE variations with BPM are plotted in Fig. 4 for all 76 breathing patterns in a real-time prediction frame. Both breathing irregularities and BMP affect prediction precision. A trend line to the 5th order polynomial is also plotted in Fig. 4. A gradual increase of prediction error with BPM for all three error rates are observed as a higher BPM is equivalent to a smaller sampling rate of the breathing waveform. Therefore, a simple solution to improve the overall prediction accuracy is to increase the position sensor sampling rate. For BMP > 18, the trend line bends downwards slightly. This can be explained as the randomness in the waveform distortion. Only a few patient breathing curves fall within the range outside a BMP > 18 so there also exists statistical uncertainty.

We have selected 15 representative motion curves with different irregularities and plotted the predictive values in BPM groups of 1–10, 10–15 and 15–18, as described in the caption to Fig. 5. All three look-ahead times are evaluated for the NMSE and MAE. In one respiration cycle, the largest error occurs near a peak or valley. Increased prediction errors are also observed near breathing irregularities that cause localized large motion displacement such as spikes. Absolute errors (AE), i.e., $AE=\left|y-{\widehat{y}}\right|$ are also plotted in Fig. 5 to monitor the real-time AE. Clinically, an AE < 1 mm for the beam exposure margin is considered outstanding, AE < 3 mm is adequate and AE < 5 mm is acceptable^43^. Three horizontal lines are plotted to mark the 1, 3, and 5 mm margins in the AE plot.

Combining all 76 time-series datasets, we have calculated the averaged prediction errors based on all predicted data values and ground truth to obtain the average NMSE = 0.025 ± 0.020, average MAE = 0.34 ± 0.20 mm and average RMSE = 0.45 ± 0.25 mm. As a comparison, Mafi et.al^20^ reported a static FNN with online retraining method that achieved RMSE = 0.69 ± 0.33 mm and a dynamics RNN that obtained RMSE = 0.57 ± 0.20 mm and MAE = 0.54 ± 0.13 mm. Tumor motion prediction of RMSE = 0.67 ± 0.36 mm and MAE = 0.57 ± 0.17 mm in a 3-layer perceptron neural network were also reported in^18^ lately. Our motion prediction results are comparable with those reported in literatures while the significance of this work lies in much reduced data processing time comparing to other machine learning approaches, the key to achieve real-time motion prediction. It is worth noting that different groups use different respiratory motion datasets^18–23^ so it is not rigorous to make direct comparison in RMSE, MAE or NMSE with other literature reported prediction results.

## Discussion

### RC for improved radiation delivery efficiency

**Figure 6:**
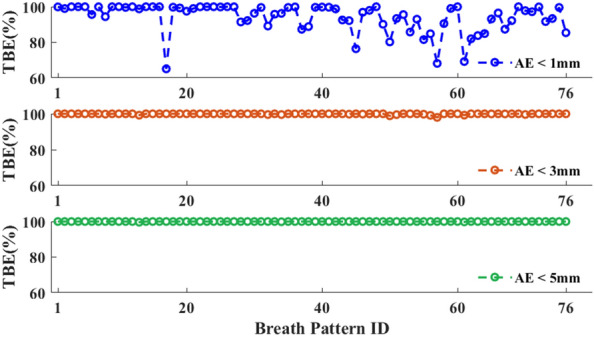
TBE for AE < 1 mm (blue), AE < 3 mm (orange), and AE < 5 mm (green). (Testing conditions: $N=14$, $N^{\prime}=12$, ${h}_{p}$ = 10, ${t}_{p}$ = 333 ms, ${k}_{tr}=600$).

In a clinical setting, the real-time photonic TDR respiratory motion prediction strategy and instrumentation described here is anticipated to be adopted in a dynamic tracking system used in combination with the respiratory gating for radiotherapy^44–46^. Radiation is synchronized with the gating window and the beam is only de-shuttered when the position prediction error is lower than a pre-set error margin. The ratio of the gating window over one respiration cycle marks the radiation treatment efficiency. NMSE, MAE, or RMSE^47^ are commonly used figures of merit of statistics. In this case, these figures of merits are used to assess machine learning outcomes while they are not explicitly used for evaluating the radiation treatment efficiency. In this work, the TBE via the time percentage of the predictive value with AE < 1, 3, and 5 mm margins within one radiotherapy session are calculated and plotted in Fig. 6. For a majority of the 76 motion curves used in this study, a TBE of 80% can be obtained for AE < 1 mm for all three look-ahead times while > 98% TBE for AE < 3 mm margin. In comparison, the current clinically implemented respiration gating method is typical to set the gating phase of 20–70%^41^. Assuming equal time in each phase, the radiation beam is on for phases from 0 to 20% and 70 to 90%, giving ~ 50% as the TBE. Using the real-time RC algorithm implemented on a photonic TDR, a significant increase in beam delivery efficiency is observed for both small and large AE margins in all three look-ahead time conditions.

### Real-time RC versus fixed training model prediction

**Figure 7:**
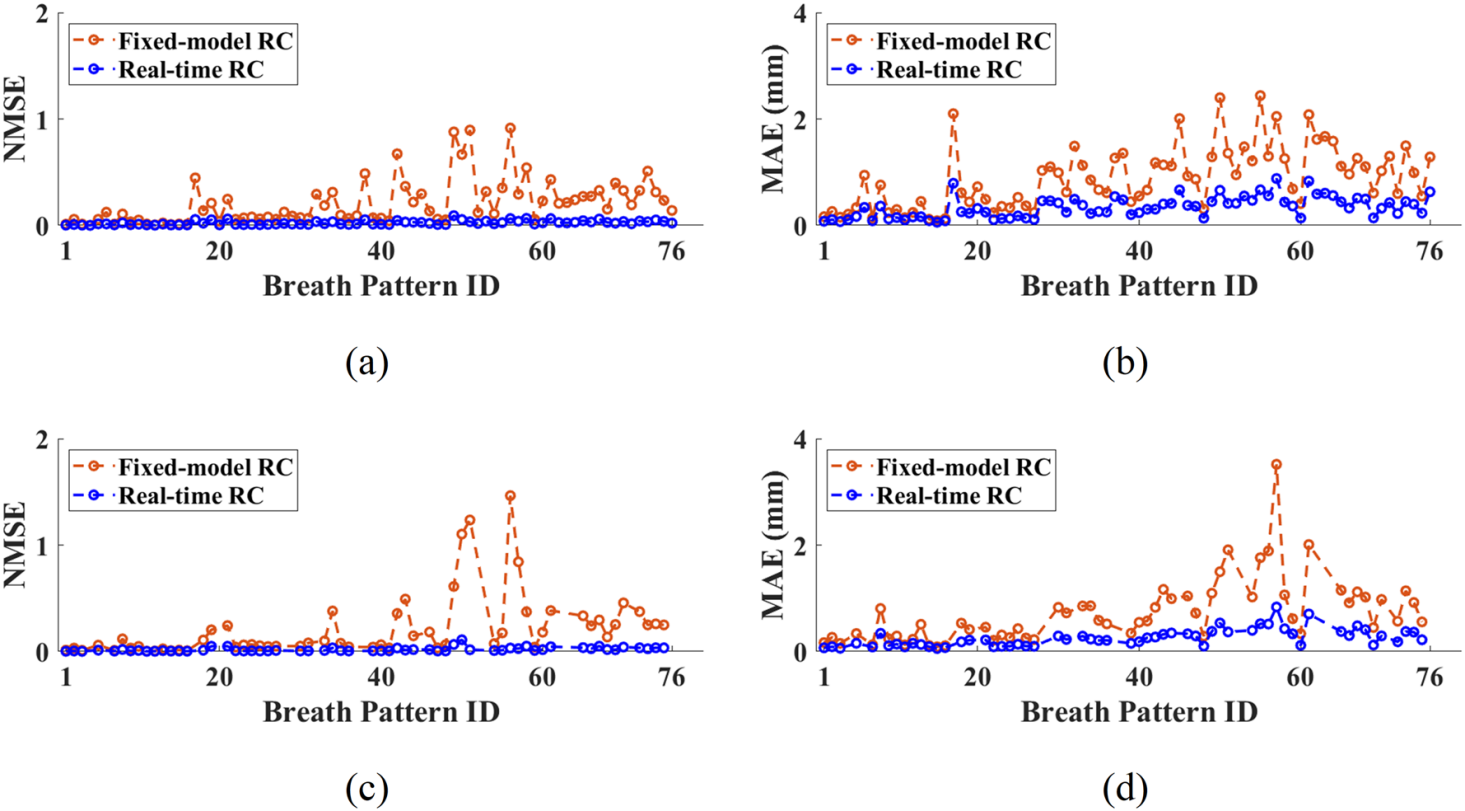
Comparison of the fixed-model RC and real-time RC motion prediction. (**a**)–(**b**): real-time RC ${t}_{tr}$ = 20 s, fixed-model RC ${t}_{tr}$ = 20 s; (**c**)–(**d**): real-time RC ${t}_{tr}$ =  0 s, fixed-model RC ${t}_{tr}$ = 60 s. (Testing conditions: $N=14$, $N^{\prime}=12$, ${h}_{p}$ = 10, ${t}_{p}$ = 333 ms).

To date, learning-based respiration motion prediction algorithms often use a pre-collected breathing pattern library to train the neural network to lift the constraint of in-line training due to large computational resource requirements. This training strategy is known to be subject to relatively larger errors as the breathing pattern characteristics tend to vary drastically among individuals or even for the same patient. This means that the respiratory motion training and testing data collected at different times is largely inadequate. Another approach is to use a portion of one breathing motion dataset for training while the remaining dataset is used for validation and prediction. In this case the training data yields a fixed ${W}_{out}$ that is used for all motion signal predication calculation. This approach is termed “fixed-model RC” in this work. For a real-time RC, ${W}_{out}$ varies as the training data window ${t}_{tr}$ (${k}_{tr}$) slides along the respiratory motion data. To compare the fixed-model RC with the real-time RC, the NMSE and MAE are calculated and plotted in Fig. 7. For the real-time RC, the sliding training signal window is selected to be ${t}_{tr}$ = 20 s (${k}_{tr}$ = 600). For the fixed-model RC, two scenarios of ${t}_{tr}$ = 20 s and 60 s (${k}_{tr}$ = 1800) are used. For the latter case, 17 breathing motion datasets having a total signal length < 1 min are excluded from the fixed-model RC calculation.

**Figure 8:**
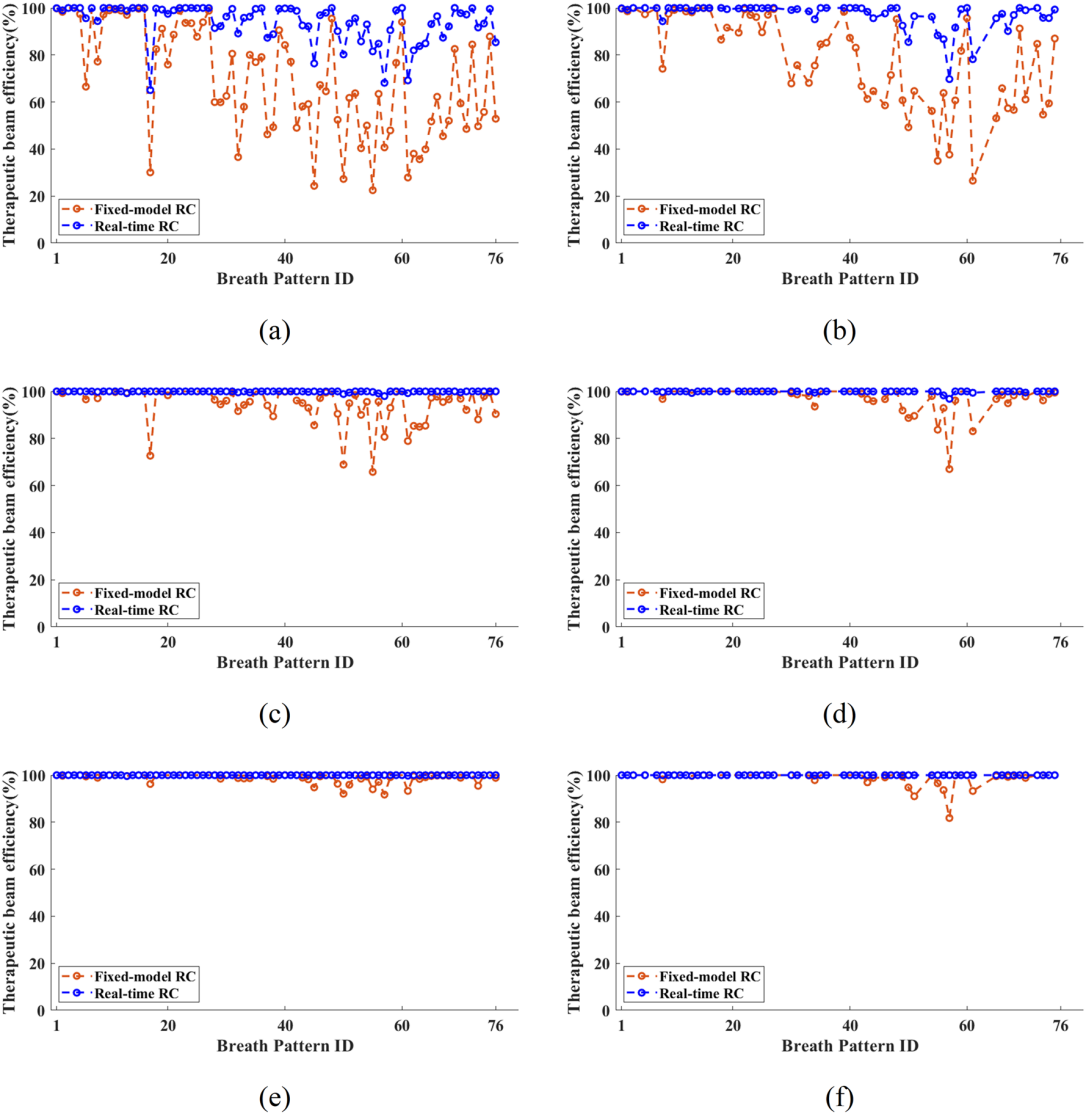
Comparison of the fixed-model RC and real-time RC motion prediction. (**a**) and (**b**) are for AE < 1 mm threshold; (**c**) and (**d**) are for AE < 3 mm threshold; (**e**) and (**f**) are for AE < 5 mm threshold. In (**a**), (**c**), and (**e**), the fixed-model RC uses a training length of 600 data while (**b**), (**d**) and (**f**) uses training length of 1800 (Testing conditions: $N=14$, $N^{\prime}=12$, ${h}_{p}$ = 10, ${t}_{p}$ = 333 ms).

TBE measures the radiation toxicity caused by the tumor respiratory movement, so an outstanding TBE is important for clinic deployment of the real-time RC. TBE for absolute error (AE) < 1 mm, AE < 3 mm and AE < 5 mm are plotted in Fig. 8 for comparison. TBE in real-time has outperformed fixed model RC for all AE scenarios in both testing cases. For a tighter AE threshold, i.e., AE < 1 mm, the improved prediction accuracy has drastically increased the TBE. For example, in Fig. 8a, only 4 out of the 76 patents have TEB < 80% using real-time RC while 44 patients have TBE < 80% for the fixed RC model.

Real-time RC has demonstrated better prediction precision in NMSE and MAE than the fixed-model RC approach for both scenarios, even though for the latter case, a longer training dataset ${t}_{tr}$ = 60 s is used for the fixed-model RC. As the distortion mechanism of the waveform formed by the peak-valley-peak pattern of the respiratory motion is highly versatile, the breathing pattern characteristics vary continuously with time but can transition rapidly to a different pattern just in a few respiratory cycles. When distortions or irregularities appear frequently in the breathing waveform, earlier motion signals become less correlated to the current breathing pattern. An enhanced class separability, reflected by the computed ${W}_{out}$ matrix can give more accurate predictive values by discarding those earlier data in the training. By updating the training model with the latest acquired position data, the real-time RC has the capability to track the pattern changes. The real-time RC also demonstrates considerably improved AE results than that of fixed-model RC, indicated by the TBE calculation.

### Real-time RC versus ridge regression classifier

Experiments were conducted to compare the RC network-based motion prediction with a simple ridge regression classifier on all 76 patient datasets. For the ridge regression classifier, the input data are directly processed in the output layer and the weight matrix ${W}_{out}$ is trained according to Eq. 5. Both NMSE and MAE are evaluated for ${k}_{w}$ = 1 to favor of smaller matrix size for fast computing; and the results are reported in Table 1 with different virtual node number. Using RC (*N* = 14, *N*′ = 12), NMSE and MAE is reduced by 45% and 3% compared to the results by ridge regression, respectively. We also tested all patient breathing curves when the reservoir node number is set much larger, i.e. *N* = 325, *N*′ = 323 to obtain richer dynamics. The NMSE and MAE is 65% and 37% lower than the ridge regression prediction results, respectively, indicating that the reservoir layer helps to improve the prediction accuracy for this motion prediction task.

**Table 1:** Comparison of predictions by real-time RC and ridge regression classifier algorithm.

Real-time RC (*N* = 14, *N*′ = 12)		Real-time RC (*N* = 325, *N*′ = 323)		Ridge regression classifier (without RC)	
NMSE	MAE (mm)	NMSE	MAE (mm)	NMSE	MAE (mm)
0.26 ± 0.12	1.28 ± 0.65	0.16 ± 0.12	0.83 ± 0.45	0.47 ± 0.33	1.32 ± 0.67

In real-time RC method, $N=14$, *N*′ = 12 are chosen for the reservoir layer. (Testing conditions: ${h}_{p}$ = 10, ${t}_{p}$ = 333 ms).

### Dynamics of delay-line reservoir

The separation property of an RC can often be improved by increasing the reservoir dynamics. It is commonly perceived that a larger number of virtual nodes in an RC will result in richer dynamics so improved class separability. Recently, a lemma from information theory that supposes the existence of an optimal reservoir node number for a given computation task, is analyzed mathematically in Ref.^34^. In the photonic TDR implementation, the virtual node number is adjusted by changing the fiber delay length, i.e., $\tau$. Without any extra fiber, the photonic TDR has an intrinsic delay time of 28 ns, corresponding to $N$ = 14 in our setting. In the experiment, we studied the inclusion of an extra fiber up to 1 km in length for extended true-time delay. A 1 km fiber corresponds to a signal delay of ~ 4.9 µs and $N$ = 619. The tested NMSE varies slightly in the range of $N$ = 14–619. As empirically the test is stable at small number of nodes, we selected $N$ = 14 with $\tau$ = 28 ns for fast RC computing.

In a physical RC, the slowest component in the reservoir oscillator determines the response time (denoted as T_D_) of a TDR. The feedback photodetector (PD) has a marked bandwidth of 1 GHz, so the response time of the TDR is comparable to $\theta$ (2 ns). The reservoir states of adjacent virtual nodes in the time domain would then interact in the form of electromagnetic wave resulting in complex transient response of the reservoir. The quantitative relation of the response time with $\theta$ was discussed in Appeltant et al.^32^ and Larger* et al.*^48^. Unsynchronized virtual nodes can also lead to enriched reservoir dynamics^5^. In this work, the node separation $\theta$ is fixed at 2 ns, choosing *N*′ = 12 resulting in a node mismatch of *k*_*N*_ = *N* − *N*′ = 2 for asynchronization. Other node mismatch conditions and how ${k}_{N}$ affects the reservoir dynamics are described in detailed in the Supplementary material.

### Computation time consideration

For the real-time RC, ${W}_{out}$ is computed and refreshed with any new motion data every ${t}_{s}$ = 33.3 ms which sets the time budget for each computing operation. In general, the input signals are sent to the RC at an input rate set by an analog-to-digital convertor (ADC) and/or the digital-to-analog converter (DAC). In this work, the sampling rate of the AWG is set at 500 MSa/s, i.e., $\theta$ = 2 ns to inject the $u(t)\cdot m(t)$ to the photonic reservoir. With a training data length of ${k}_{tr}$ = 600 and $N$ = 14, the signal processing time is ~ 14.4 µs (${k}_{tr}\times N^{\prime}\times \theta$). The full system computation time and the alternative approach in realizing the hardware-based RC for real-time implementation are discussed in the Supplementary material.

## Conclusion and future works

In this paper, we explored respiratory motion prediction using a real-time RC model based on a photonic time-delayed hardware system. It demonstrates that a small training dataset of 600 data points are sufficient to train the RC network for various breathing patterns. A smaller training dataset leads to reduced matrix size so that RC computing time can be significantly reduced. Improved prediction accuracy was demonstrated compared to the fix-model RC approach as well as the simple ridge regression classifier. The limitation of the proposed real-time RC lies in the difficulty to adjust the hardware setting, i.e. tuning the hyperparameters based on each patient’s breathing curve whereas the lack of hardware adaptability can cause decreased prediction accuracy. In the future, we will study the predictive AE quantitatively and assess how critical to introduce individually optimized RC network for each patient.

## Supplementary Information

Supplementary Information.

## Data Availability

The datasets used and/or analyzed during the current study are available upon request to the corresponding author.
