# Supplementary material for: Real-time respiratory motion prediction using photonic reservoir computing
Source: Sci Rep. 2023 Apr 7;13:5718. doi: 10.1038/s41598-023-31296-2 (PMC10082218; doi:10.1038/s41598-023-31296-2)
Supplement: Supplementary file 1 — Supplementary Information. [file 41598_2023_31296_MOESM1_ESM.pdf]

Supplementary Information

Real-time respiratory motion prediction using photonic reservoir computing

Zhizhuo Liang[1], Meng Zhang[1], Chengyu Shi[2], and Z. Rena Huang[1,*]

[1]Rensselaer Polytechnic Institute, Troy, New York 12180, United States

[2]City of Hope Medical Center, Duarte, California 91010, United States
[*] Corresponding author: huangz3@rpi.edu

Gaussian filter to reduce motion data noise

In this tumor motion prediction task, the tumor motion data sometimes show random perturbation and spikes due to breathing irregularities which can increase the prediction difficulty. To lower the impact of those irregularities in tumor motion patterns, a Gaussian smoothing filter is applied to the motion data before the motion data are processed in the photonic reservoir computer. The smoothing process is implemented by weighted average of the data that follow a 1-D Gaussian distribution:

$$G(x) = \frac{1}{\sqrt{2\pi}\sigma} e^{-\frac{x^2}{2\sigma^2}} \quad \text{(s1)}$$

where σ is the standard deviation which quantifies the amounts of smoothing. By applying this filter to the tumor motion data, the sharp and sudden changes of the curves are smoothed, and high-frequency noise components are greatly reduced. In this experiment, different σ values from 0.5 to 12 are tried and σ is eventually chosen to be 7.5, where the smallest error rates are reported.

Breathing pattern classification

For most patients, the inhalation phase, an active physical motion, is longer than the exhalation phase, a passive motion. Reflecting on the breathing patterns (BP), we expect uneven durations when signals rise and fall. In the ideal case, the breathing pattern follows a periodic quasi-sinusoidal curve. For many lung cancer patients whose lung functions are compromised, breathing patterns would be expected to exhibit large variations from normal breathing pattern curves. Several common breathing patterns with irregularities are shown in Fig. s1. Fig. s1 (a) is a normal breathing pattern as it has uniform breathing rate and amplitude. Typical irregularities include base-line shift [Fig. s1 (b)], amplitude change at intervals of approximately t = 10 - 20 seconds [Fig. s1 (c)], spikes at intervals of approximately t = 130 seconds [Fig. s1 (d)], staircase curve due to short breathing gasps [Fig. s1 (e)] and some combination of multiple irregularities [Fig. s1 (f)].

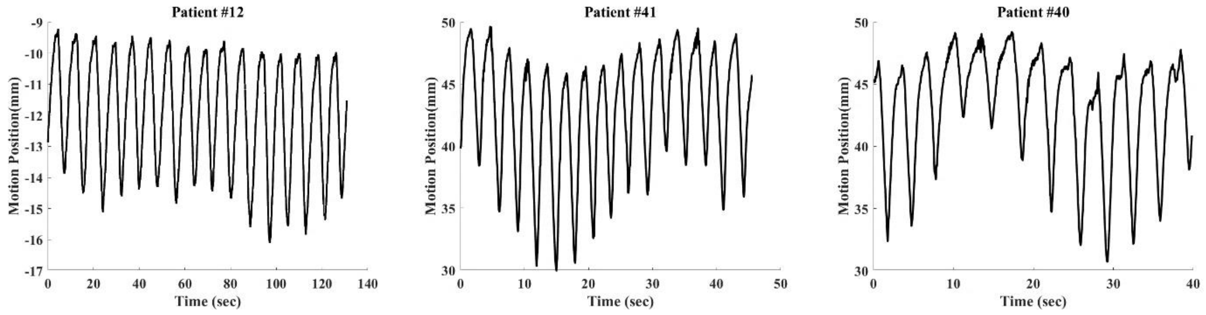

(a) **(b)** **(c)**

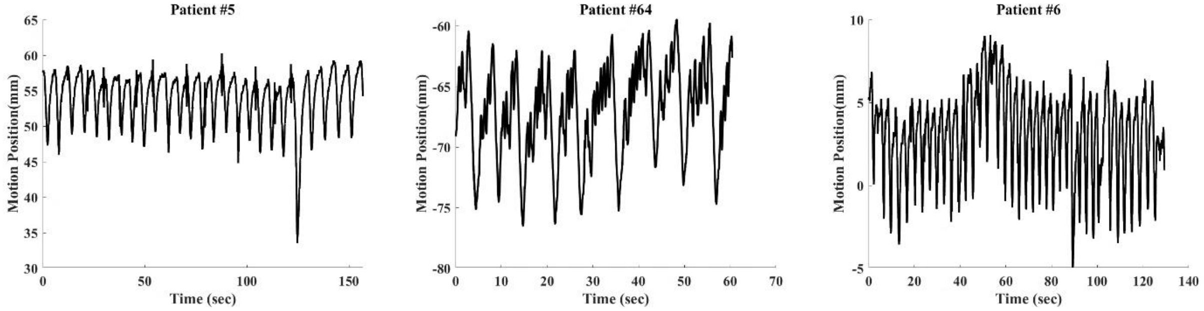

(d) **(e)** **(f)**

Fig. s1: Several typical examples of irregular breathing patterns that lead to versatile temporal motion curves taken under conditions that the patient is in a quiet and relaxed state for at least 15 minutes prior as confirmed by slow breathing, low blood pressure and low heat rate. (a) A relatively normal breathing curve with even rate and uniform amplitude; (b) baseline shift typically caused by patient slow upper body position movement during radiation treatment; (c) an example of gradual amplitude variation over time; (d) an example of the abrupt occurrence of a pulse with large amplitude likely caused by an involuntary event such as sneezing; (e) staircase curve superimposed on a quasi-sinusoidal function, likely due to a patient with compromised lung function; and (f) a combination of more than one irregularity: baseline shift and large amplitude variation.

Hyper-parameters optimization

In our photonic reservoir computing system, a continuous wave (CW) light at wavelength of 1.55 μm passes through a Mach-Zehnder modulator (MZM) to produce optical signals modulated by a time-dependent voltage signal $V(t)$. For an optical input I_0 to MZM, the time-dependent output intensity $I(t)$ after the MZM is given by

$$I(t) = I_0 \cos^2\left[\frac{1}{2}\left(\frac{\pi V(t)}{V_\pi} + \varphi_0\right)\right] = \frac{1}{2} I_0 \left[1 + \cos\left(\frac{\pi V(t)}{V_\pi} + \varphi_0\right)\right] \quad \text{(s2)}$$

where φ_0 is the operational set point phase shift produced by an adjustable DC bias voltage, $V_{DC,}$ and is given by

$$\varphi_0 = \varphi_{offset} + \frac{\pi V_{DC}}{V_\pi} \quad \text{(s3)}$$

where φ_{offset} is the initial MZM phase offset.

In Eq. s2, the time-dependent voltage, $V(t)$, includes the information of both previous data signals after a round trip and the current input data signals. Let A be the product of the photodetector (PD) responsivity $\mathcal{R}$ [A/W] multiplied with the PD's effective resistance, R, given by (the PD's intrinsic + an external) resistance in [Ohm]. Then A will be in unit of [V/W].

The dimensionless parameter, η_O, marks the retained ratio after the signal completes one roundtrip pass over a delay-line optical loop, including 90/10 splitter and fiber-optical coupler, while η_E is the retained ratio after the electrical signal traverses the entire electrical path, which includes an electrical combiner and an electrical amplifier. A detailed expression of $V(t)$ is

$$V(t) = \eta_O A \eta_E I(t - \tau) + \eta_E V_{AWG} u(t) \quad \text{(s4)}$$

where τ is the round-trip time, V_{AWG} is the maximum arbitrary waveform generator (AWG) output voltage and $u(t)$ is the relative amplitude of the signals normalized to [-1, 1], injected to the reservoir layer from the input layer. As we measure the light intensity, i.e., the product of photon energy multiplied with the number of photons per second per unit area rather than the amplitude, $I(t)$ ranges from 0 to I_0. To make the time evolution equation dimensionless, we define the normalized intensity $x(t) = \frac{2I(t)-I_0}{I_0}$, also known as the dimensionless reservoir state and rescale it to [-1, 1]. The time evolution equation then becomes

$$x(t) = \cos\left[\frac{\pi\eta_O A\eta_E I_0}{2V_\pi} x(t-\tau) + \frac{\pi\eta_E V_{AWG}}{V_\pi} u(t) + \varphi_{offset} + \frac{\pi V_{DC}}{V_\pi}\right]. \quad \text{(s5)}$$

Comparing equation s5 with the general form $x(t) = \sin[\alpha x(t-\tau) + \beta u(t) + \varphi]$ obtained from equations s2 and s3 we can obtain the three key parameters - feedback gain α, input gain β and reservoir bias of φ. These are defined as follow:

Feedback gain: $\alpha = \frac{\pi\eta_O A\eta_E I_0}{2V_\pi}$

Input gain: $\beta = \frac{\pi\eta_E V_{AWG}}{V_\pi}$

Bias: $\varphi = \varphi_{offset} + \frac{\pi V_{DC}}{V_\pi} + \frac{\pi}{2}$ (s6)

In this study, the extracted feedback gain α and β values are $\alpha = 0.87$, $\beta = 2.29$ based on the measurement results.

Virtual nodes and networks dynamics

Computation time and motion prediction precision are two key performance indicators for this real-time forecasting task. For the photonic RC, the virtual node separation θ is set experimentally by the AWG sampling rate. When the period of the pre-set masking function τ' is smaller than the delay loop time τ, the network is working in unsynchronized regime and connections among neighboring nodes are formed to increase the reservoir dynamics. The optical intensity in the reservoir that contains input data information gradually decreases due to the short-term memory property of the reservoir. Hence, the current reservoir state of one node depends on the reservoir states of other nodes, contributing to richer dynamics[1,2].

With the delay loop time τ measured, the virtual node separation time θ and the masking period τ' experimentally set, N (the maximum number of nodes that can be used for readout) and N' (the number of virtual nodes that produce reservoir response) follow the relations below

$$\tau = N\theta \quad \text{(s7)}$$

$$\tau' = N'\theta \quad \text{(s8)}$$

$$N = N' + k,\ \ k \in \{0,1,2,\dots,N-1\} \quad \text{(s9)}$$

where k is the node mismatch between N and N'. When $k = 0$, the system works in the synchronized regime, i.e., $N' = N$. When the system works in unsynchronized regime, a new parameter k' is defined to describe the node connection offset among the N' nodes that are used for readout. For the non-zero, but small k scenario, i.e., $0 < k < N/2$, which is the case most widely discussed in previous works in the unsynchronized regime[1,2], the node connection offset of this N'-node system k' is identical to k, $k' = k = N - N'$ and the reservoir states in discrete time can be described as

$$x_i(n) = \sin(\alpha x_{i-k'}(n-1) + \beta m_i u(n) + \varphi), \qquad k' \le i < N' \tag{s10}$$

$$x_i(n) = \sin(\alpha x_{N'+i-k'}(n-2) + \beta m_i u(n) + \varphi), \qquad 0 \le i < k' \tag{s11}$$

where n represents the discretized time and i is the node index. For all the N' nodes that are used in this case, i.e., the reservoir states of $N' - k'$ nodes, interact with the reservoir states of the previous period, i.e., $n-1$, and the reservoir states of the remaining k' nodes interact with those from 2 periods ago, i.e., $n-2$. In this work, θ is experimentally set at 2ns and $\tau = 28\text{ns}$ is measured, so with τ' chosen as 24ns. Then N and N' can be easily calculated and found to be $N = 14$ and $N' = 12$, leading to a node mismatch $k = 2$. The corresponding network can be described by equation (s10) and (s11). However, when $k \ge N/2$, i.e., $N' \le N/2$, less than half of the maximum virtual nodes are used, meaning that the reservoir states of each node will interact with past reservoir states that pertain to at least the previous two periods. In this case equation (s10) and (s11) no longer precisely describe the node connections. To generalize equation (s10) and (s11) so that they can be applicable to any $k \in \{0,1,2,\dots,N-1\}$, a new parameter j is defined to characterize the number of past rounds of the reservoir states that interact with the present reservoir state. The parameter j can be obtained by calculating the quotient of N/N':

$$N/N' = j \dots k', \qquad k' \in \{0,1,2,\dots,N'-1\} \tag{s12}$$

The remainder k' is just the node connection offset parameter we defined before. When $N' \le N/2$, k' is no longer equal to k. The time-evolution equation (s10) and (s11) can thus be generalized as

$$x_i(n) = \sin(\alpha x_{i-k'}(n-j) + \beta m_i u(n) + \varphi), \qquad k' \le i < N' \tag{s13}$$

$$x_i(n) = \sin(\alpha x_{N'+i-k'}(n-j-1) + \beta m_i u(n) + \varphi), \qquad 0 \le i < k' \tag{s14}$$

Equation (s10), (s11) are special cases of equation (s13), (s14). When $j = 1$, Equations (s13) and (s14) become equation (s10) and (s11). A comparison between two models with different node mismatch k are illustrated in Fig. 2. The nodes in yellow effectively produce reservoir response in experiments for readout while those in gray do not. The virtual nodes are connected sequentially, forming a ring topology. The dashed arrows illustrate how the internal states of each node interact with one another from the past rounds. The input signals travel counterclockwise (from a larger node index to a smaller node index) in two loops shown in Fig. 2. The system with $N' = 12$ and $k = 2$ is shown in Fig. 2(a), where the system node # 2 - # 11 have the present reservoir states interacting with the past reservoir states of the last round (marked by τ'). For nodes # 0 and # 1, the present reservoir states interact with the earlier reservoir states in 2 past rounds (marked by $2 \times \tau'$). Fig. 2(b) shows the system with $N' = 3$, $k' = 2$, $j = 4$, suggesting that the nodes interaction with feedbacks from at least 4 periods. To be more specific, node # 2 has the current state interacting with states from 4 periods ago ($4 \times \tau'$, blue dashed arrow), and for nodes # 0 and # 1, the interactions occur with internal states from 5 periods ago ($5 \times \tau'$, yellow and red dashed arrows). Using $n = 6$ as an example, the three current reservoir states $x_i(6)$, i.e., the internal states for $i = 0, 1, 2$ can be expressed as

$$x_0(6) = \sin(\alpha x_1(1) + \beta m_0 u(6) + \varphi)$$

$$x_1(6) = \sin(\alpha x_2(1) + \beta m_1 u(6) + \varphi)$$

$$x_2(6) = \sin(\alpha x_0(2) + \beta m_2 u(6) + \varphi) \tag{s15}$$

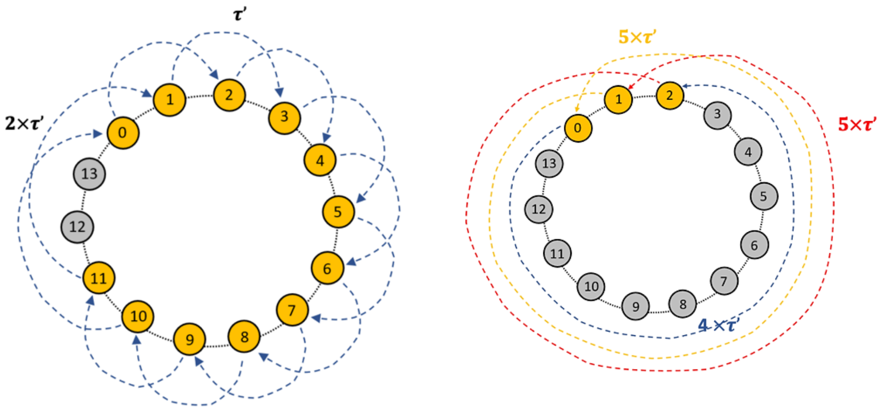

(a) (b)

Fig. s2: Two models of an N=14 virtual node interconnection (a) $N' = 12$, $k = k' = 2$, $j = 1$, with small node mismatch k; (b) $N' = 3$, $k = 11$, $k' = 2$, $j = 4$ in large misalignment between N and N' and the node mismatch is $k = 11$ while the node connection offset for this 3-node system is $k' = 2$.

As discussed above, complex signal connection/interaction can be built by choosing small N' values for richer temporal dynamics. To explore the impact of different node mismatch N', data sets of three patients were studied: BP #8, 55, 65 based on breathing rate and tested with different node mismatch under the same hardware condition ($N = 14$). The results are shown in Fig. s3. The computation time can be significantly decreased as the virtual number N' becomes smaller while the prediction remains accurate.

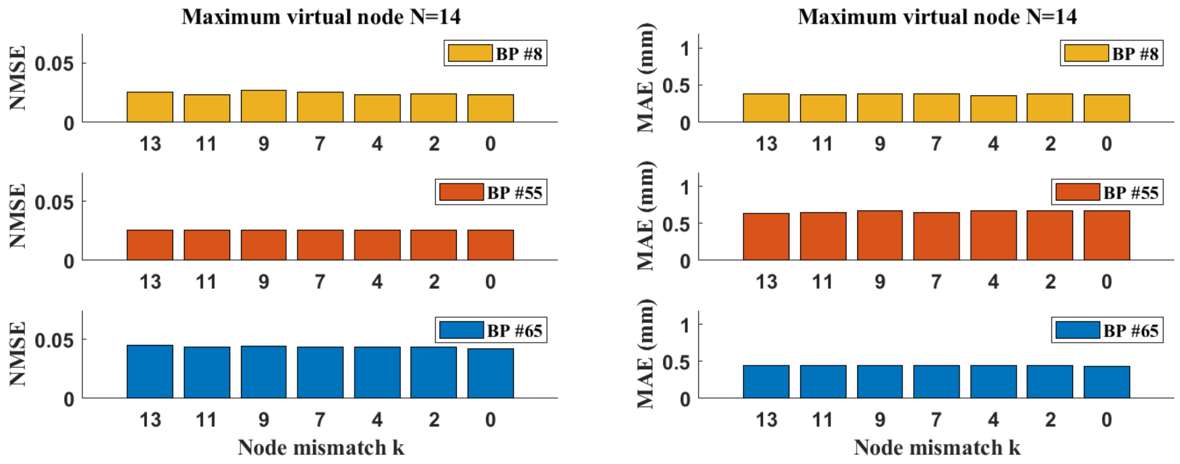

(a) (b)

Fig. s3: Respiratory motion prediction (a) NMSE and (b) MAE of BP #8(slow), 55(normal), 65(fast) as a function of node mismatch. In this study, the maximum virtual node number N is set as $N = 14$. (Testing conditions: $N = 14$, $h_p = 10$, $k_{tr} = 600$)

For the case of large node mismatch such as $k = 13, N = 14,$ and $N' = 1$, there is only one effective virtual node in the reservoir. Node connection analysis indicates that the reservoir response of this single virtual node will be influenced by the reservoir response of the initial signal from 14 periods before ($14 \times \tau'$). In this case, a richer temporal dynamic is achieved without spatial interactions among virtual nodes, similar to the case of $k = 0, N = 14,$ and $N' = 14$ in which there is no asynchronization so the signals on all 14 nodes only interact with the prior reservoir response produced 1 period earlier ($1 \times \tau'$). In

the latter case, there is also no spatial connections among all virtual nodes so a reservoir with temporal dynamics only can also form delay-line RC network.

Additional Photonic TDR Computation Time Consideration

The photonic TDR hardware settings such as the modulator bias, fiber length, loop gain are configured so that satisfactory forecasting results can be obtained for all motion signal curves. At present, both the input layer and output layer signals processing are carried on an off-line computer. The 90/10 optical splitter taps out 10% of the optical signal power to a readout photoreceiver (Optilab PR-12-B-M). The reservoir states $x(n)$ can be displayed and stored in an oscilloscope (Keysight MSOS404A) and later processed off-line for the motion signal training of the neural network and output prediction. Although, in this work, signal processing of the input and the output layer are carried out on an off-line computer, analogous photonic signal processing has been reported by Duport et al.[3]. Briefly, cascaded MZMs are used to perform matrix multiplication $u(t) \cdot m(t)$ in the optical domain for the input signals while a balanced photoreactor and a balanced modulator are combined to process both negative and positive data values in the output layer. The time delay due to optical signal propagation across the multiple RC layers constructs the characteristic time of the physical RC. Time delay from the photonic analogue hardware of the input and output layer are mainly caused by the limited bandwidth of each component, estimated to be on the order of nanoseconds. In this work, the reservoir has a measured delay time of 28ns therefore, the total RC physical layer characteristic time is on the order of tens of nanoseconds.

The most time-consuming step is the inverse matrix calculation to determine W_{out} during the training phase. The reservoir state matrix X has a dimension of $N' \times k_{tr}$ while the computation time increases exponentially with size of matrix X. In this work, the real-time motion prediction algorithm is programmed in MATLAB runs on a desktop with an Intel i7 8700 8-core CPU. The processing time is ~ 150 μs. A field-programable gate array (FPGA) is an appealing alternative for online training to minimizing interface data flow latency. Antonik et al.[4] reported a gradient descent training algorithm running on a Xilinx ML605 FPGA, with an execution time of 2.2ms for N = 50.

Motion Sensor Data Collection

Respiratory motion curves were acquired using a Varian system (RPM, Varian Medical Systems, Palo Alto, CA, USA). The patients were supine laying down on the computer tomography (CT) table and were instructed to breathe freely and relax. The RPM system has an infrared reflective box placed near the patient’s chest or abdominal region and an infrared camera to capture the motion of the patient’s surrogate changes based on the box's reflected light. The system measures the patient’s respiratory pattern and range of motion, collectively displayed as a waveform which captures the amplitude and phase of the breathing cycle as a function of time and saves the data into the CT system. The saved data can be used by the CT system to further process the 4D CT based on either amplitude or phase binning.

Incorporate RC to adaptive radiation therapy

There are various ways that a photonic RC for motion prediction can be incorporated in adaptive radiation therapy. Conventional tracking, real-time tracking and predictive tracking were proposed for clinical adoption. Detailed discussion can be found in[5,6].

Radiator Latency consideration

There is a system latency in adjusting the multi-leaf collimator in a radiation system, which is caused by the combined software and hardware delay. The latency, a critical system variable in determining the look-

ahead time of the RC algorithm, varies in a large range among different radiation system vendors. Vero, RadiXact, and Unity systems report a latency of 48 ~ 70 ms [7,8,9], CyberKnife system has 115 ms[10], and TomoTherapy's motion-adaptive delivery system has a latency from 175 ms up to 500 ms[11,12]. A long latency time of several hundreds of milliseconds is needed to account for the differences in tumor sizes and shape. In this work, for a general study, we focus on short to medium range of equipment latency, i.e., the motion prediction look-ahead time of 66.6 ms, 166.6 ms and 333.3 ms.

References

1. Paquot, Y. *et al*. Optoelectronic reservoir computing. *Sci. Rep.* **2**, 287 (2012).

2. Duport, F., Schneider, B., Smerieri, A., Haelterman, M. & Massar, S. All-optical reservoir computing. *Opt. Express* **20**, 22783-22795 (2012).

3. Duport, F., Smerieri, A., Akrout, A., Haelterman, M. & Massar, S. Fully analogue photonic reservoir computer. *Sci. Rep.* **6**, 22381 (2016).

4. Antonik, P. *et al*. Online training of an opto-electronic reservoir computer applied to real-time channel equalization. *IEEE Trans. Neural Net. Learn. Syst.* **28**(11), 2686-2698 (2017).

5. Eom, J., Shi, C., Xu, X. G. & De, S. Modeling respiratory motion for cancer radiation therapy based on patient-specific 4DCT data. in *Lecture Notes in Computer Science (including subseries Lecture Notes in Artificial Intelligence and Lecture Notes in Bioinformatics)* vol. 5762 LNCS (2009).

6. Guo, B., George Xu, X. & Shi, C. Real time 4D IMRT treatment planning based on a dynamic virtual patient model: Proof of concept. *Med Phys* **38**, (2011).

7. Depuydt, T. *et al.* Treating patients with real-time tumor tracking using the Vero gimbaled linac system: Implementation and first review. *Radiother. Oncol.* **112**(3), 343-351 (2014).

8. Schnarr, E. *et al.* Feasibility of real-time motion management with helical tomotherapy. *Med. Phys.* **45**, 1329-1337 (2018).

9. Depuydt, T. *et al.* Geometric accuracy evaluation of the new VERO stereotactic body radiation therapy system. *UKACC Int. Conf. on Control*, **8,** 259-264 (2010).

10. Seppenwoolde, Y., Berbeco, R. I., Nishioka, S., Shirato, H. & Heijmen, B. Accuracy of tumor motion compensation algorithm from a robotic respiratory tracking system: A simulation study. *Med. Phys.* **34**, 2774-2784 (2007).

9. Lu, W. Real-time motion-adaptive delivery (MAD) using binary MLC: II. Rotational beam (tomotherapy) delivery. *Phys. Med. Biol.* **53**, 6491-6511 (2008).

12. Bedford, J. L. *et al.* Effect of MLC tracking latency on conformal volumetric modulated arc therapy (VMAT) plans in 4D stereotactic lung treatment. *Radiother. Oncol.* **117**(3), 491-495 (2015).
